# Two sisters reveal autosomal recessive inheritance of epidermodysplasia verruciformis: a case report

**DOI:** 10.1186/1471-5945-14-12

**Published:** 2014-07-21

**Authors:** Rui Yoshida, Toshihiko Kato, Masahiko Kawase, Mariko Honda, Tsuyoshi Mitsuishi

**Affiliations:** 1 Department of Dermatology, Nippon Medical School, 1-1-5, Sendagi, Bunkyo-ku, Tokyo 113-8603, Japan; 2 The Research Institute of Vaccine Therapy for Tumors and Infectious Diseases, Nippon Medical School, 1-1-5, Sendagi, Bunkyo-ku, Tokyo 113-8603, Japan; 3 Department of Dermatology, The Jikei University School of Medicine, 3-25-8, Nishi Shinbashi, Minato-ku 105-8461, Tokyo, Japan; 4 Department of Dermatology, Tokyo Women’s Medical University Yachiyo Medical Center, 477-96, Ohwada-Shinden, Yachiyo 276-8524, Chiba, Japan

**Keywords:** Epidermodysplasia verruciformis, Hereditary pattern, Human papillomavirus, *EVER1* and *EVER2* genes, Electron microscopic examination

## Abstract

**Background:**

Epidermodysplasia verruciformis is a rare genodermatosis characterized by a unique susceptibility to cutaneous human papillomaviruses infection. Most patients show autosomal recessive patterns of inheritance.

**Case presentation:**

We report a case of two sisters with clinically epidermodysplasia verruciformis specific lesions on the face, neck, trunk, and extremities. PCR analysis indicated the presence of human papillomavirus type 5 in the lesions. Electron microscopic examination showed viral-like particles in keratinocyte nuclei and the stratum corneum of the epidermodysplasia verruciformis lesions. In addition, we examined the *EVER1* and *EVER2* genes using eight different primer pairs without finding any nonsense or frameshift mutations in the gDNA from lymphocytes of the elder sister.

**Conclusions:**

In this report, the patient’s parents did not have epidermodysplasia verruciformis lesions or a consanguineous marriage. EV did not develop in the elder sister until five years of age, so the parents did not perceive EV as an inherited disease. The probability that EV developed in both sisters was only 6.25%. Thus, it is rare for both sisters to develop epidermodysplasia verruciformis lesions considering that the parents were presumed to be carriers and the disease reveal an autosomal recessive pattern of inheritance.

## Background

Epidermodysplasia verruciformis (EV) is an uncommon cutaneous disorder characterized by persistent infection with *beta*-human papillomavirus (HPV) and a combination of flat wart-like lesions, pityriasis versicolor-like lesions, hypopigmented macules, or other erratic skin lesions. These lesions are mainly located on the face, neck, and extremities [1,2]. Most patients demonstrate autosomal recessive patterns of inheritance, although some exhibit X-linked recessive or autosomal dominant inheritance [3,4]. Cutaneous carcinomas in situ or invasive carcinomas, which are usually of the Bowen’s disease-type, generally appear in a high percentage of patients, before the age of 40 [1,2,5]. More than 40 *beta*-papillomaviruses have been identified in patients with EV [6]. Among them, HPV5 and HPV8 are primarily involved in the malignant transformation associated with EV. HPV9 is also often involved in EV [1,2].

The *EVER1* and *EVER2* genes were found to be responsible for EV in 2002 [7]. *EVER1* and *EVER2* are also referred to as *TMC6* and *TMC8,* respectively, and are located on chromosomes 17q25 [7]. Various mutations in the *EVER1* and/or *EVER2* genes reportedly contribute to HPV-associated EV [8-10]. In this study, we report a case of two sisters with both EV and HPV5 infections whose parents did not have EV lesions. We observed viral-like particles in the lesions by electron microscopic examination. We also assessed the mutational status of the *EVER1* and *EVER2* genes from extracted blood cells using PCR with eight different primers.

## Case presentation

### Case 1

**Figure 1:**
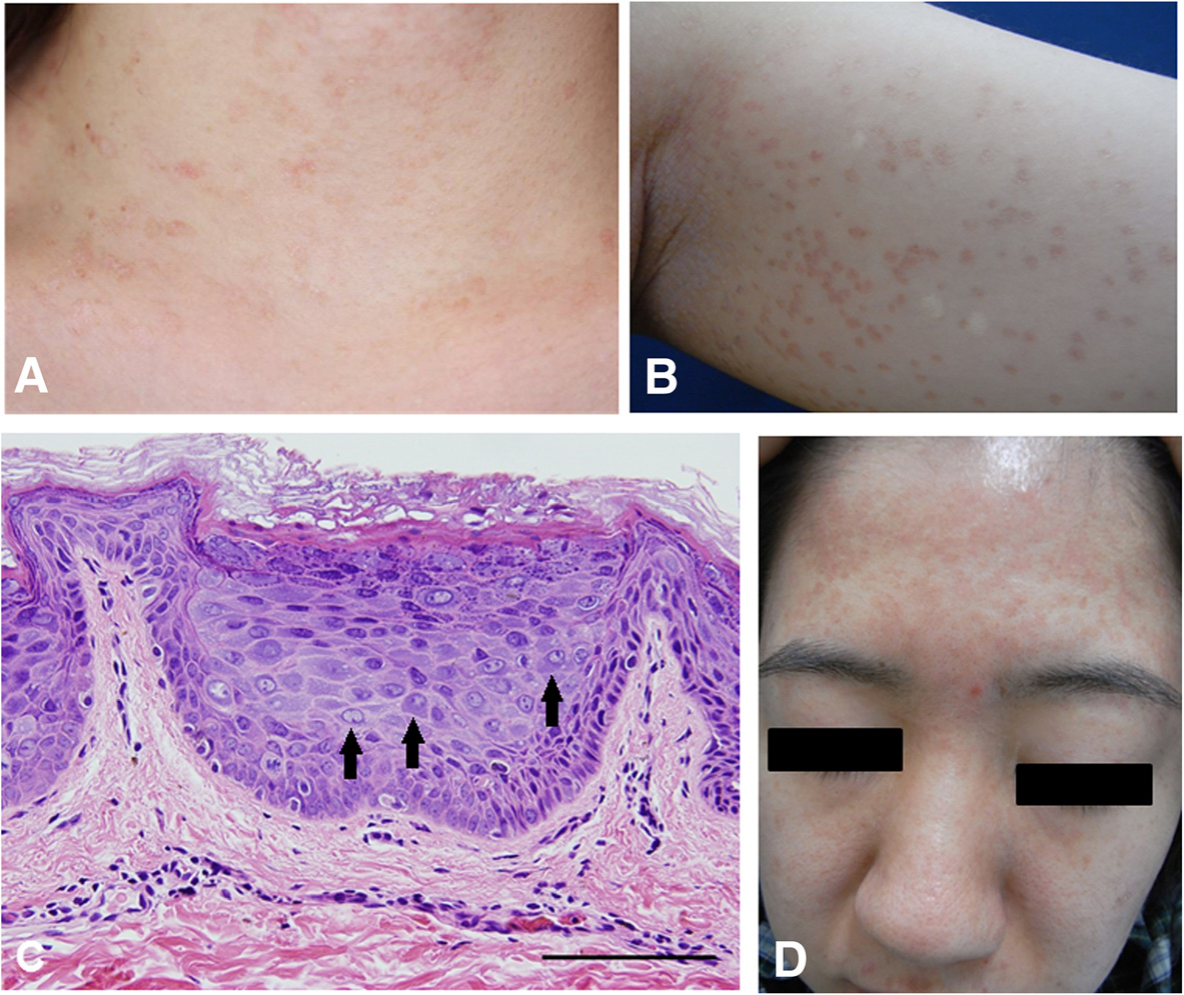
**Clinicohistological findings in two sisters. (A)** Asymptomatic, slightly erythematous, and hypopigmented pityriasis versicolor-like lesions on the neck in the elder sister. **(B)** Left upper arm of the elder sister showing numerous round pigmented macules with slight scale. **(C)** Histological findings show epidermal hyperplasia and distinct homogenous intracytoplasmic inclusion bodies in the large clear cells of the epidermis (arrows); scale bar = 100 μm. **(D)** Multiple brown, flat wart-like lesions on the face of the younger sister.

A 29-year-old female has had asymptomatic, slightly erythematous, and hypopigmented pityriasis versicolor-like lesions on her face, neck, trunk, and extremities since the age of five as well as multiple brownish-black, hyperkeratotic, papular plaque, wart-like lesions on the extremities. The lesions progressively increased in number and size with age (Figure 1A, B). No abnormalities were observed upon examination of the hair, nails, mucosal membranes, and other systems, including abdominal echography and chest X-ray. Histopathology of a flat wart-like lesion showed marked hyperkeratosis, mild acanthosis, and the presence of distinct homogeneous intracytoplasmic inclusion bodies in the large clear cells of the epidermis (Figure 1C). Additional systemic examinations and laboratory investigations, including an HIV test, were all normal. Topical 5-fluorouracil (5-FU) and imiquimod 5% cream were applied to the small pityriatic or flat wart-like lesions for three days per week at Jikei University School Hospital and Nippon Medical School Hospital. The lesions gradually decreased in size and number. She has been applying ultraviolet (UV) blocker most days and is being followed by Nippon Medical School Hospital.

### Case 2

The 23-year-old sister of case 1 has had similar cutaneous lesions since the age of six. Multiple asymptomatic, erythematous, pityriasis versicolor-like lesions and flat wart-like lesions were found on the face, trunk, and extremities (Figure 1D)*.* These lesions also increased progressively in number and size. A biopsy of the EV lesions showed focal cellular atypia in the flat wart-like lesions with abnormal mitotic figures in the large clear cells of the epidermis. Topical 5-FU and imiquimod 5% cream were prescribed for application twice daily on the lesions. She has been applying UV blocker almost every day. Interestingly, neither parents of the two patients showed EV lesions, but both were carriers.

### Genetic investigations

We performed PCR using two different primers, CP65/70 and SK, to determine the prevalence of HPV infection [11,12]. Frozen tissues from the EV cutaneous lesions of both patients were digested with proteinase K (100 mg/ml), and genomic DNA (gDNA) was extracted with phenol-chloroform-isoamyl alcohol. The gDNA was then precipitated with ethanol as previously described [12]. The primer sets amplified DNA products from *alpha*-, *beta*-, and *gamma*-HPVs. The PCR products were sequenced with an ABI model 3730xl automated DNA sequencer (Applied Biosystems, Foster City, CA, USA) according to the manufacturer’s instructions. The result of the PCR analysis indicated that HPV5 infection was present in the flat wart-like lesions and pityriasis versicolor-like lesions from the lesions in both sisters.

After informed consent was obtained, gDNA from the peripheral blood of the elder sister was acquired according to the manufacturer’s instructions. The presence of *EVER1* and *EVER2* mutations was analyzed by PCR with six known primers [8-10] and two new primers, which were designed against GenBank sequences (NM_152468) for *TMC8/EVER2*. The new primers used for amplification of the gDNAs were as follows: *EVER2*, exon 8-1 F, 5’-GTG GAG CTG GAG GAG GG-3’; exon 8-1R, 5’-TCC TTG TTG TCC TGT GAG TAC TTG-3’; exon 8-2 F, 5’-GTT TCC TGC ACC CTT TCC TC-3’; exon 8-2R, 5’-TAC TTG GTA GCC CAG AAG ATG G-3’. The PCR products from the two new primers were 170 bp and 211 bp in length, respectively. The PCR products were run on 2% agarose gels and purified with the QIAquick Gel Extraction Kit (Qiagen, Hilden, Germany). Sequencing analysis was carefully performed in both directions and compared with the known sequences registered in GenBank. We did not identify any nonsense or frameshift mutations in any of the eight target bands.

### Electron microscopic examination

**Figure 2:**
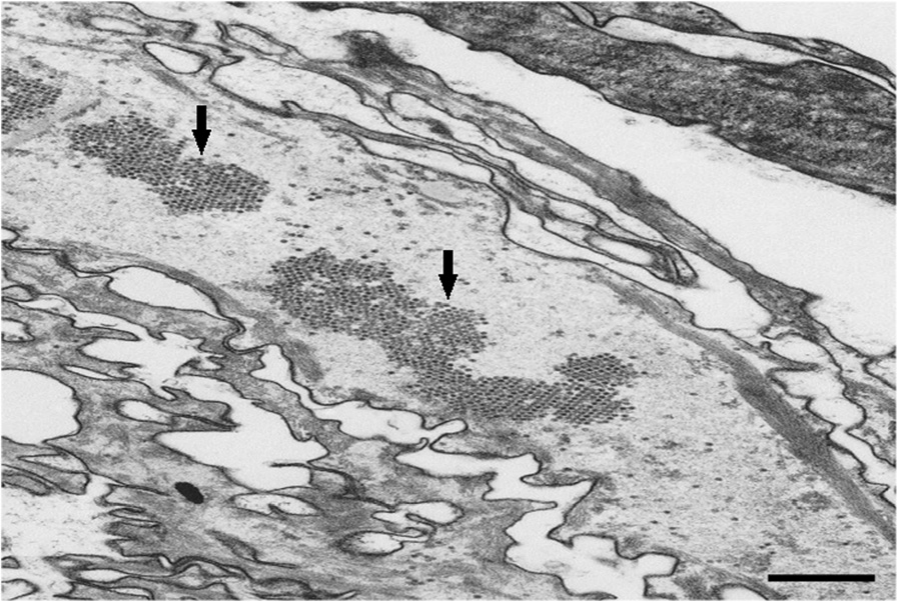
Electron microscopy image showing typical virus-like particles with crystalline array (arrows) filling the stratum corneum; scale bar = 1 μm.

Electron microscopy was performed on a lesion from the skin of the elder sister. The specimens were fixed with 2.5% glutaraldehyde and postfixed with 1% osmium tetroxide. The samples were then dehydrated through a graded alcohol series and embedded in Epon 812. Ultrathin sections were cut using an Ultracut N Ultramicrotome (Reihert-Nissei, Tokyo, Japan) and were stained with uranyl acetate and lead citrate. The sections were then examined by electromicroscope (Hitachi H-7500; Hitachi, Tokyo, Japan). The results of the electron microscopy showed typical virus-like particles with crystalline arrays filling the nuclei of the keratinocyte cells in the upper epidermis and stratum corneum. Large, clear dysplastic cells were also observed in the flat wart-like lesions (Figure 2).

## Discussion

Malignant cutaneous lesions in patients with EV are preferentially located on sun-exposed areas. The development of malignant transformation in EV patients is primarily associated with HPV5 and HPV8 infection. PCR analysis indicated that HPV5 infection was present in flat wart-like lesions and pityriasis versicolor-like lesions of our patients; however, malignant cutaneous lesions were not found in sun-exposed areas. Most patients have autosomal recessive inheritance patterns for EV. However, a small number of patients show X-linked recessive or autosomal dominant inheritance [3,4]. The parents of the patients in our study were presumed to be carriers, but did not develop EV lesions. In addition, the parents did not form a consanguineous marriage. Based on autosomal recessive patterns of inheritance, we would expect 25% of children to develop EV lesions. In this family, these sisters are the only offspring, and the mother did not have a history of miscarriage. EV did not develop in the elder sister until five years of age. Thus, the parents did not identify EV as a disease of inheritance.

The *EVER1 and EVER2* genes associated with EV belong to the transmembrane channel-like (*TMC*) gene family and are called *TMC6* and *TMC8*, respectively. The albuminous functions encoded by *EVER1* and *EVER2* remain unknown. The presence of *EVER* gene mutations in patients with EV suggests that *beta-*HPV infection is linked to the progression of non-melanoma skin cancer. Some reports indicate the relationship between mutations in the *EVER2* gene and the risk of cutaneous squamous cell carcinoma [13,14]. After careful examination for the presence of *EVER1* and *EVER2* genes by PCR followed sequence analysis in both directions, we did not observe any nonsense or frameshift mutations in any of the target bands. Although there is no curative therapy for EV, protection from UV light and treatment of EV-specific cutaneous lesions is necessary from an early stage. Nonsurgical approaches include topical imiquimod, 5-FU, vitamin D3, cimetidine, systemic retinoids, and interferon [14-18]. Some reports show success with topical imiquimod in immunocompetent patients with squamous cell carcinoma *in situ*[14,19]. In this study, imiquimod 5% cream was applied once per day and three days per week for flat wart-like lesions. Oral retinoids (0.5 mg/kg) were also prescribed. Cutaneous lesions decreased in size after a short period; however, relapse of the cutaneous lesions of patients occurred after 1 year, suggesting that the therapy was not effective. Importantly, both patients applied UV blocker daily.

## Conclusions

Most EV patients show autosomal recessive patterns of inheritance; although, some patients can exhibit X-linked recessive or autosomal dominant inheritance. In the two cases described here, two sisters with EV had parents without EV lesions that were are not in a consanguineous marriage. Furthermore, the mother does not have a history of miscarriage. EV did not develop in the elder sister until five years of age*.* The probability of EV developing in both sisters was only 6.25%. Thus, it is rare for both sisters to develop EV lesions, despite that both parents were presumed to be carriers and the disease showed an autosomal recessive pattern of inheritance. No nonsense or frameshift mutations in the *EVER2* gene were identified using two new primers, however, there may be some risk of developing cutaneous squamous cell carcinoma from benign EV lesions. Long-term follow-up will be necessary for both sisters.

## Consent

Written informed consent was obtained from the patients for publication of this Case report and any accompanying images. A copy of the written consent is available for review by the Editor of this journal.

## Abbreviations

EV: Epidermodysplasia verruciformis; HPV: Human papillomavirus; UV: Ultraviolet; gDNA: Genomic DNA.

## Competing interests

The authors declared that they have no competing interests.

## Authors’ contributions

RY, MK, MH, and TM analysed clinical and pathological data, including those from patient follow up. TK, and TM performed laboratory work and helped with data analysis. RY, and TM drafted the manuscript and designed the case-study. All authors read and approved the final manuscript.

## Pre-publication history

The pre-publication history for this paper can be accessed here:

http://www.biomedcentral.com/1471-5945/14/12/prepub
